# Inverse heat transfer problem solution of sounding rocket using moving window optimization

**DOI:** 10.1371/journal.pone.0218600

**Published:** 2019-06-24

**Authors:** Adam Dąbrowski, Leszek Dąbrowski

**Affiliations:** 1 Department of Mechanics and Mechatronics, Faculty of Mechanical Engineering, Gdansk University of Technology, Gdańsk, Poland; 2 Department of Mechine Design and Motor Vehicles, Faculty of Mechanical Engineering, Gdansk University of Technology, Gdańsk, Poland; Northeast Electric Power University, CHINA

## Abstract

An Inverse Heat Transfer Problem is solved for a sounding rocket module given its geometry and measured temperature profile. The solution is obtained via moving window optimization, a technique for solving inverse dynamics. An analysis is performed to modify the method to avoid oscillatory behavior of the resulting heat flux profile. The method parameters are tuned in relation to characteristic phases of the flight. Results are presented and correlated with measured flight data. Conclusions are drawn for better experiments for measuring heat flux on a sounding rocket skin.

## Introduction

Determining heat transfer on a rocket is a crucial part in sounding rocket and payload design. For that reason, thermocouples are embedded usually into rocket skin and their data saved or transmitted to the ground station. An investigation of heat flux on the rocket skin was performed both with wall conduction neglected [1] and considered [2]. A careful design of a temperature measurement system is important to obtaining precise results [3]. These measurements are used to calculate heat flux distribution.

This poses a classical inverse problem where effect (here, temperature distribution in space and time) is given, and cause (heat flux distribution) is determined. An extensive research into inverse problems have been made in last years providing practical tools for engineering analysis [4–7]. These have been applied to inverse heat transfer problems by Beck et al. [8], Alifanov et al. [9, 10], Ozisık et al. [11] and Woodbury [12].

Several inverse heat transfer analyses of a rocket has been performed [2, 13]. Some difficulties have been pointed out, such as IHTP high sensitivity to small uncertainties in temperature measurements. A source of such errors is the thermocouple installation itself [14]. Additionally, Perakis and Haidn [15] point to response delays as a difficulty in using thermocouple measurements for determining heat flux.

The presented work provides results of inverse heat transfer analysis of a REXUS rocket. This was a sounding rocket with student experiments dedicated to various environmental measurements and technological demonstrators [16]. One experiment, SMARD, consisted of thermistors embedded in thick aluminum bulkhead [17]. Although the setup rig was not designed to determine heat flux distribution, the data was used to analyze if such calculations are possible.

## 1 Experiment

**Fig 1:**
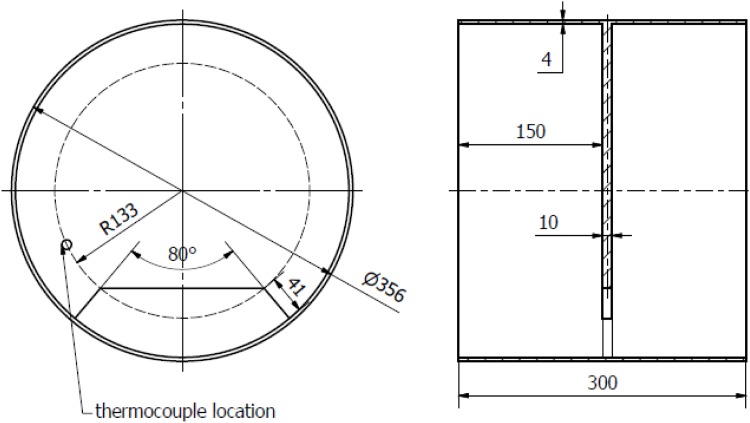
CAD drawing of the rocket module with the location of the temperature sensor marked.

The problem involves a thick aluminum bulkhead with a hole connected to a cylindrical aluminum rocket. This bulkhead is mounted in the middle of a REXUS sounding rocket. This geometry is presented in Fig 1. The sounding rocket followed a parabolic trajectory in order to reach microgravity. It reached an apogee of 81.4 km at 140 s after lift-off. Flight duration *t*_*flight*_ was 386 s.

**Fig 2:**
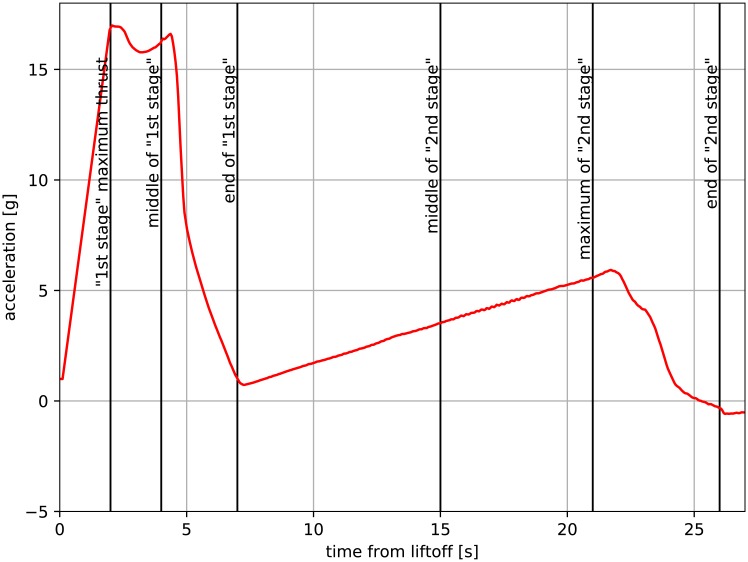
Acceleration of REXUS-18 [17].

The REXUS sounding rocket is powered by an Improved Orion Motor with 290 kg of fuel. While it is a single stage solid propellant, due to grain geometry [18] the thrust curve actually shows a two-stage behavior (see Fig 2).

During the flight, heat flux is present on the rocket skin, due to its fast ascent through the atmosphere. The heat flux is assumed to be axisymmetric on the rocket skin. This assumption is justified due to the rocket spinning (≈ 4 Hz) through most of the flight. Furthermore, uniform distribution of heat flux on rocket skin was assumed. This is justified for the payload compartment of a sufficiently long sounding rocket, as it was in this case.

A NTC type thermistor was used to measure the air (used for determining the coefficient of convection) and bulkhead temperature. These were only powered during short measurement periods to prevent measurement errors due to self-heating. The thermistors were calibrated in the thermal vacuum chamber of the Lehrstuhl für Raumfahrttechnik at TU Munich [17].

**Fig 3:**
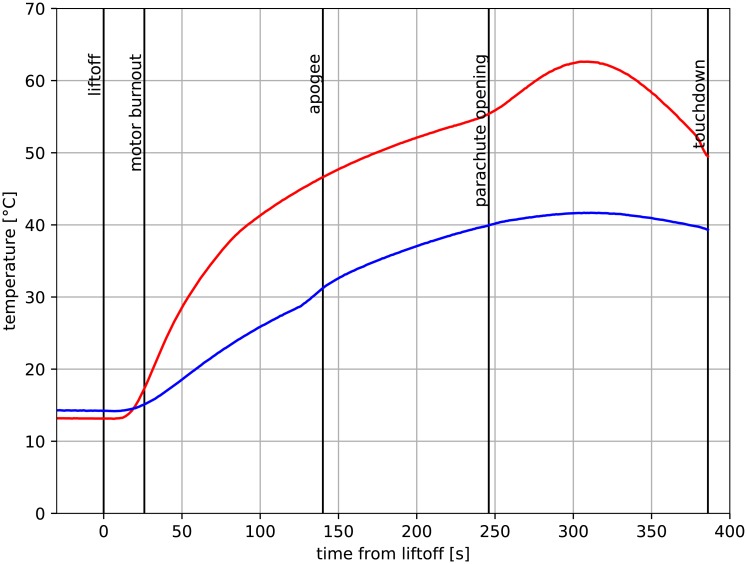
Temperature measured during flight [17].

The temperature profiles measured during the rocket flight are presented in Fig 3. Important events, such as lift-off, apogee, max. deceleration, etc. are marked with vertical lines. A complete list of these events is provided in Table 1 It can be noticed that at certain moments linked with these events, the slope of the graph changes. Such a setup was used to solve inverse heat transfer problem (IHTP), i.e. calculate the heat flux profile.

**Table 1:** Characteristic phases of flight.

time	symbol in Fig 17	phase
0 s		lift off
2 s	(a)	maximum thrust of “1st stage”
4 s	(b)	middle of “1st stage” burn
7 s	(c)	start of “2nd stage” burn
15 s	(d)	middle of “2nd stage” burn
21 s	(e)	maximum thrust of “2nd stage”
26 s	(f)	burnout of motor
70 s	(g)	entrance into mesosphere
140 s	(h)	apogee
220 s	(i)	egress from mesosphere
246 s	(j)	parachute opening
386 s	(k)	touchdown

## 2 Methods

### 2.1 Optimization methodology in an inverse problem

Generally, inverse problems are solved by minimizing an objective function with some stabilization technique used in the estimation procedure. When the transient readings *Y*_*j*_ taken at times *t*_*j*_, *j* = 1, ⋯, *N* of a single sensor are used, the problem can be treated as minimization of a function [19]:

$$\begin{array}{c} \underset{q}{min}(\sum_{j=1}^{N}|T_{j}\left(q\right)-Y_{j}\left|\right) \end{array}$$ (1)

As IHTP is an *ill-posed* problem, multiple solutions are possible. When using the optimization approach, a solution can be found that shows oscillatory behavior. Such solutions are to be avoided, as there is no physical phenomenon behind such oscillations, only computational peculiarity. An extensive list of IHTP methods was provided in [8, 20, 21].

These can be divided into exact and approximate solutions. Exact solutions usually involve a simple geometry such as cylinders or spheres, either hollow or solid [22] with well defined boundary conditions. While these give insight into inverse heat transfer, they do not act as an engineering tool for more complex geometries, such as that of a rocket.

Approximate solutions include single future time step method [23], where the heat flux component is sequentially calculated. This can be also extended to multiple temperature measurements. Another approach is the function specification method [8] where functional form of heat flux is assumed. Parameters of the function are calculated either for the whole domain or sequentially. A method used in the presented work is a modification of Beck’s function specification method. Table 2 provides description of all symbols used throughout this work.

**Table 2:** Nomenclature used in this work.

symbol	description
*κ*	coefficient of thermal conductivity
*ρ*	density
Δ*T*	average temperature error
*c*_*p*_	specific heat capacity
*Fo*	Fourier number
*h*	coefficient of heat transfer
*i*	iterative index (1 ≤ *i* ≤ *n*)
*j*	iterative index (1 ≤ *i* ≤ *N*)
*k*	length of moving window
*L*	size of FEM element
*n*	number of heat flux samples
*N*	number of temperature samples
**q**	vector of *n* heat flux values
**q**_**smallest**_	heat flux distribution in smallest finite element
*q*_*i*_	heat flux value in time *s*_*i*_
*Q*_*t*_	heat generated via aerodynamic resistance
*Q*_2_	heat flowing to the inside of the rocket
*Q*_1_	heat transported with air flow around the rocket
**s**	vector of *n* timestamps corresponding to *q*
**t**	vector of *N* timestamps corresponding to *T* and *Y*
*t*_*s*_	step time used in FEM
*t*_*flight*_	time of flight
*t*_*r*_	reaction time
**T**	vector of *N* calculated temperatures
*T*_*j*_	calculated temperature value in time *t*_*i*_
**T**_**smallest**_	temperature distribution in smallest finite element
**w**	moving window; vector of *k* heat flux values; **w** ⊂ **q**
**Y**	vector of *N* measured temperatures
*Y*_*j*_	measured temperature value in time *t*_*i*_
*T*	function describing spatial and temporal distribution of temperature
*x*, *y*, *z*	directions defining orthogonal coordinate system
*n*_*o*_	direction locally normal to outer surface of the rocket
*n*_*i*_	direction locally normal to inner surface of the rocket
*n*_*b*_	direction locally normal to boundary surface of the rocket
**B**	bulk temperature

### 2.2 Transient FEM calculations

#### 2.2.1 Model setup and time step determination

**Fig 4:**
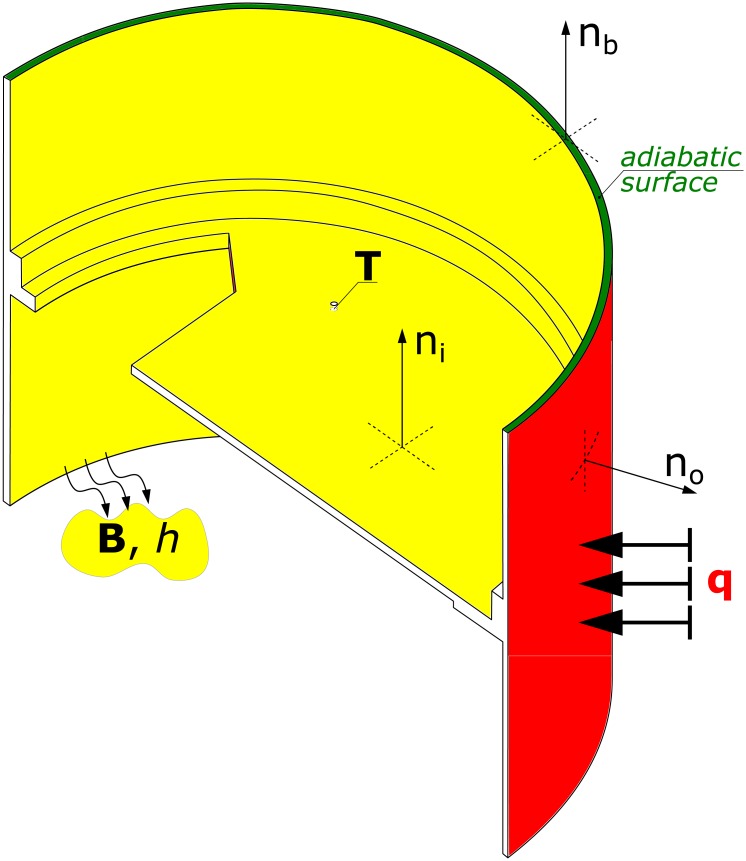
Physical model of rocket module.

In this work, the rocket module was modelled as presented in Fig 4. The heat flow can be described by heat equation:

$$\begin{array}{c} \frac{\partial }{\partial x}(\kappa \frac{\partial T}{\partial x})+\frac{\partial }{\partial y}(\kappa \frac{\partial T}{\partial y})+\frac{\partial }{\partial z}(\kappa \frac{\partial T}{\partial z})=\rho c_{p}\frac{\partial T}{\partial t} \end{array}$$ (2)

At the outer surface (red in Fig 4), a heat flux *q* is present (*n*_*o*_ is the direction locally normal to outer surface):

$$\begin{array}{c} -\kappa \frac{\partial T}{\partial n_{o}}\left(t\right){|}_{outersurface}=q \end{array}$$ (3)

The inner surface (yellow in Fig 4) is subject to convection, discussed in section 2.2.2 (*n*_*i*_ is the direction locally normal to inner surface).

$$\begin{array}{c} -\kappa \frac{\partial T}{\partial n_{i}}\left(t\right){|}_{innersurface}=h[T_{\infty }-T(t){|}_{innersurface}] \end{array}$$ (4)

It is assumed that similar rocket modules are located above and below the module described, so no heat flow is assumed through boundary surface (green in Fig 4, *n*_*b*_ is the direction locally normal to boundary surface):

$$\begin{array}{c} -\kappa \frac{\partial T}{\partial n_{b}}\left(t\right){|}_{boundarysurface}=0 \end{array}$$ (5)

Temperature *T* at thermistor location is sampled at discrete times and stored in vector **T**. Note that this temperature values **T** is then compared with measured values **Y** in optimization procedure, see Eq 1. Since the problem is solved numerically, both **T** and **Y** are vectors of samples *T*_*j*_ and *Y*_*j*_, respectively for a given time *t*_*j*_.

**Fig 5:**
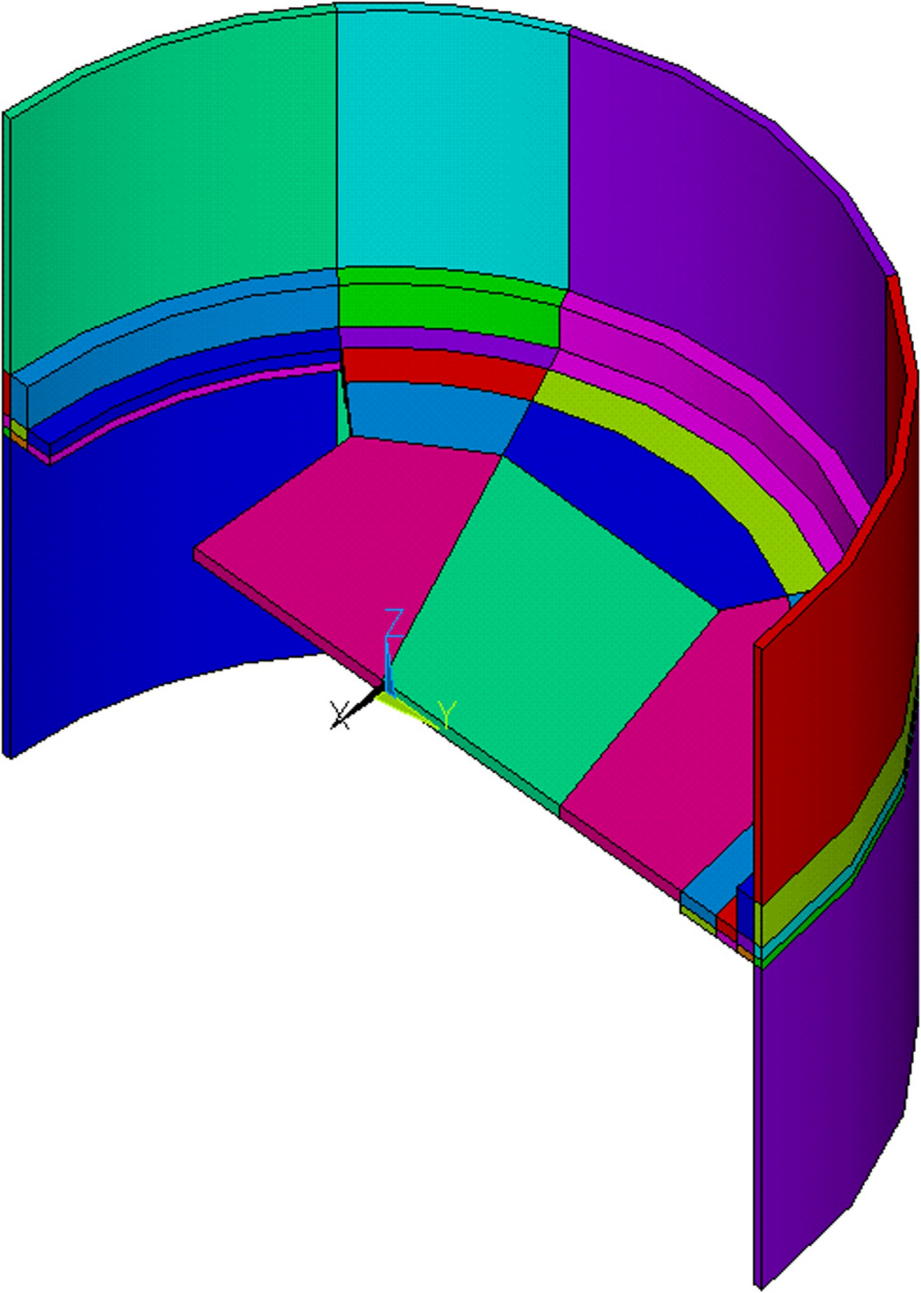
Finite element model with visible sizes of elements (number of element divisions per line equal to 1). These were the biggest elements considered and were subsequently used in the optimization procedure.

**Fig 6:**
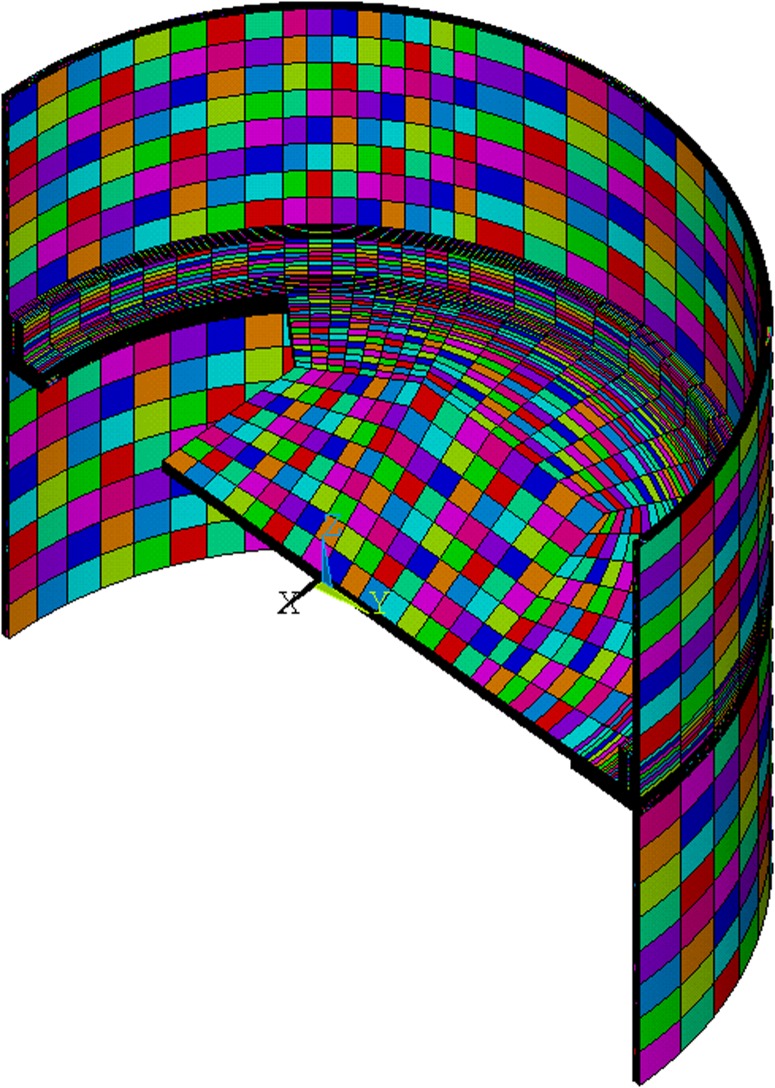
Finite element model with visible sizes of elements (number of element divisions per line equal to 8). These were the smallest elements considered and were used only to analyse element size influence on the solution.

**Fig 7:**
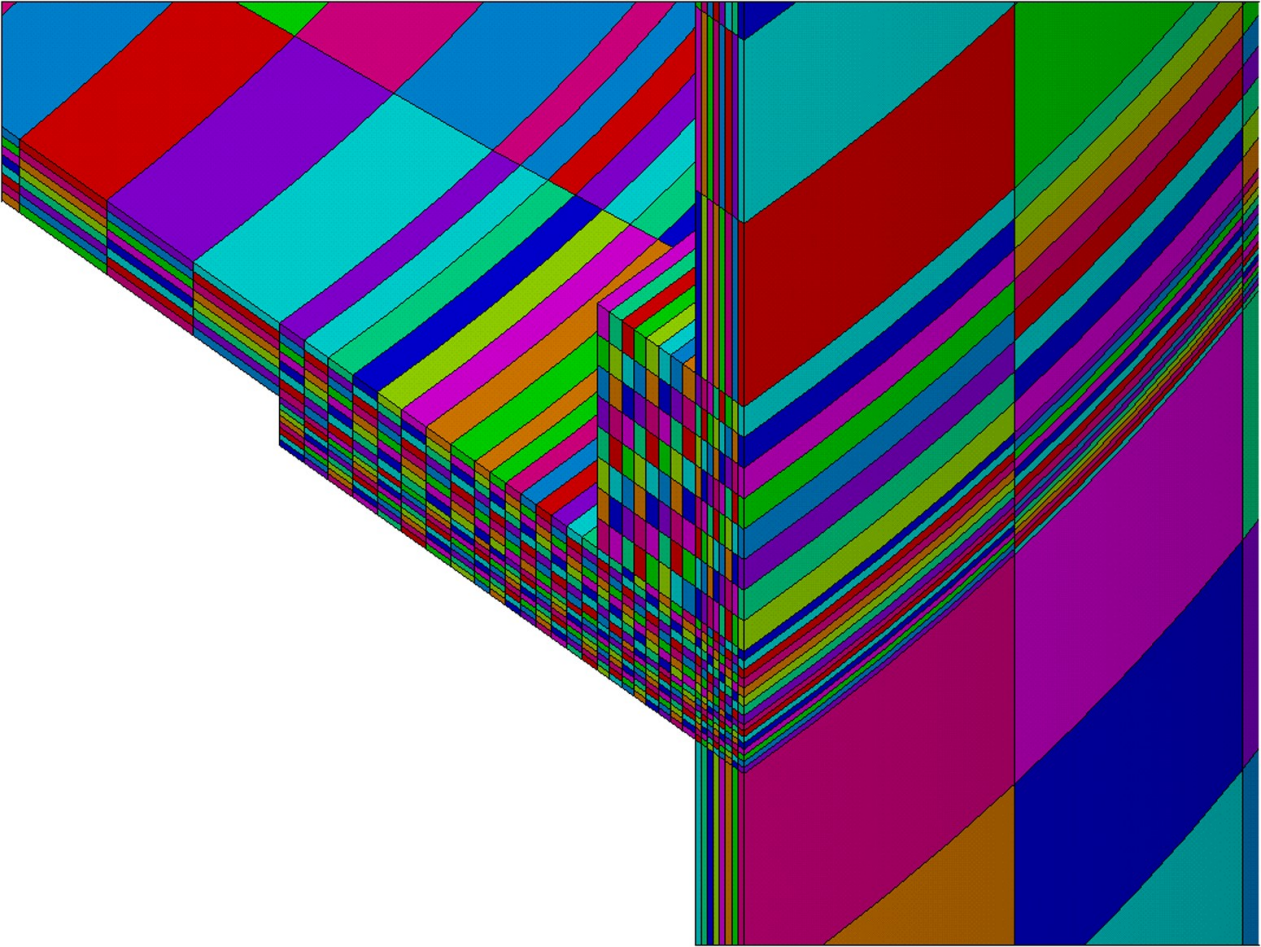
Finite element model with visible sizes of elements (number of element divisions per line equal to 8), magnified to show smallest elements.

In order to find the relation **T(q)**, the finite element method was used. Solid elements were used (see Figs 5, 6 and 7), and it was verified that element size has little influence on solution precision (see 2.2.3). For the Eq 2 parameters, Al7075 properties were used, shown in Table 3. Calculations were performed in ANSYS 18 using a 3D 20-node hexahedral thermal solid element SOLID90.

**Table 3:** Chosen material properties of Al7075 [24].

parameter	value
thermal conductivity *κ*	130 W/(m⋅K)
specific heat capacity *c*_*p*_	960 J/(kg⋅K)
density *ρ*	281 kg/(*m*^3^)

Special considerations were made while defining element size and time step. Because optimization was performed for coarsest elements meshing (number of element divisions per line equal to 1, shown in Fig 5), the smallest element size was defined as *L* = 4 mm, which is the thickness of the rocket skin. A practical value of length of time step *t*_*s*_ = 1 s was chosen. This is the same as the temperature measurement **Y** sampling frequency, which allows a direct comparison of the measured temperature **Y** and calculated temperature **T** profiles. The Fourier number can be calculated for this case, according to the following formula:

$$\begin{array}{c} Fo=\frac{\kappa }{c_{p}\rho }\frac{t_{s}}{L^{2}} \end{array}$$ (6)

In the case of this FEM model, the largest Fourier number value was *Fo* = 30.1. This then fails to satisfy the criterion *Fo* ≫ 0.20 [25] (or *Fo* ≫ 0.25 [26]), therefore, an additional analysis of the numerical stability of the solution was performed.

#### 2.2.2 Boundary conditions

The outer surface was loaded with an initial time-dependent value of uniform heat flux **q**. The internal surfaces were constrained with convection where the bulk temperature was changed with a frequency of 1 Hz according to values measured by [17].

Determining the coefficient of heat transfer *h* is a problematic task [2]. As the rocket is not sealed, a complete outflow of air from inside occurs during the flight. This fluid motion with respect to the aluminum elements is greater when considering the time-dependent acceleration of the rocket. These effects would result in convective heat transfer of unknown properties. However, a rapid pressure drop eventually decreases convection to zero. The authors adopted value *h* = 5 W/(*m*^2^K) as a speculation (the typical value for air and aluminum interface free convection is 20 W/(*m*^2^K) [24]). Tests showed that varying the value of *h* between these two values had an insignificant effect on temperature profiles. In such difficult cases of heat transfer, the calibration of heat transfer coefficient *h* is achieved by means of optimization [27].

**Fig 8:**
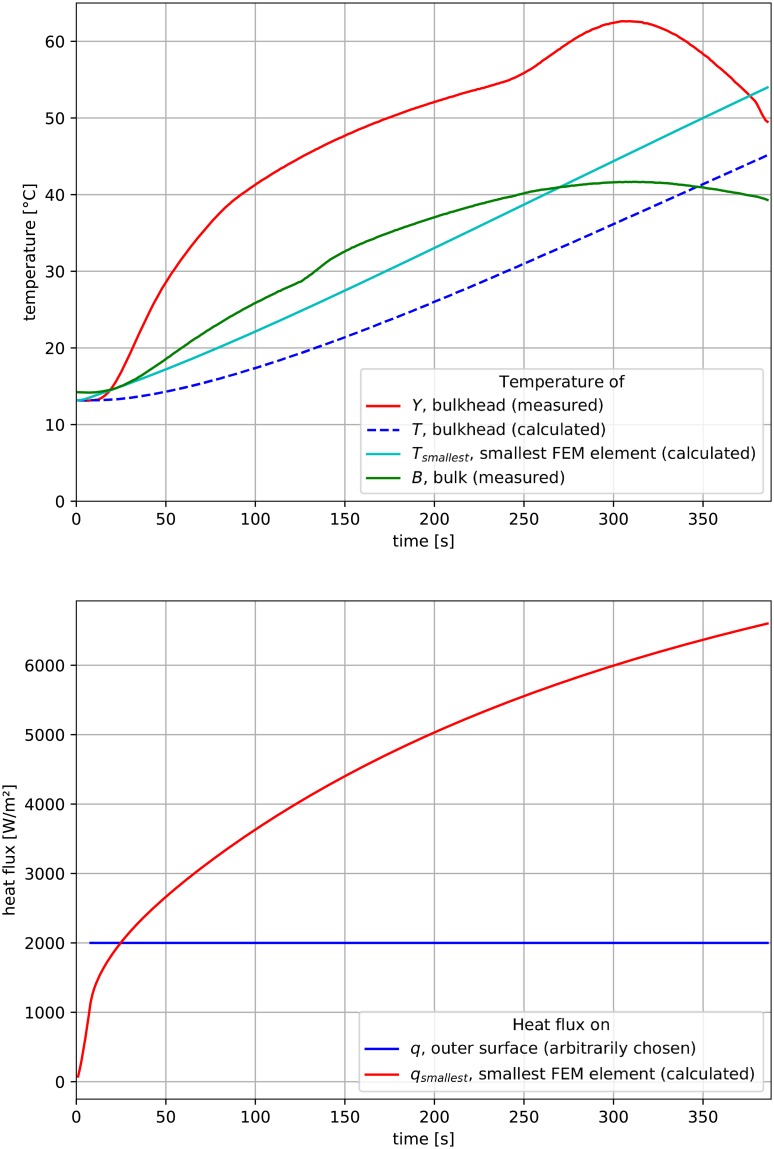
Results of simulations of heat flow subject to constant heat flux.

A constant heat flux **q** ≡ 2000 W/*m*^2^ was chosen, for which the corresponding temperature distribution **T** was of the order of measured temperature **Y**. These were presented in Fig 8 (**q**—bottom, blue curve; **T** top, blue dashed line; **Y** top, red line).

#### 2.2.3 Tests

IHTP procedure described in subsection 2.3 required several thousands of goal function calls (see Table 4), i.e. direct heat transfer problem solutions, to reach a required accuracy (stop criterion described in section 2.3.2). These solutions were obtained using FEM model with biggest finite elements (smallest number of elements per line *N*_*DIV*_ = 1, presented in Fig 5). The computation time of the optimization procedure using Tryton supercomputer [28] took over three days nonetheless.

**Table 4:** Comparison of optimization results for test with various moving window lengths.

window length *k*	total goal function calls	average error (7) Δ*T* [°*C*]
1	463	0.259
2	970	0.069
3	1666	0.051
4	2636	0.048
16	3022	0.635

**Fig 9:**
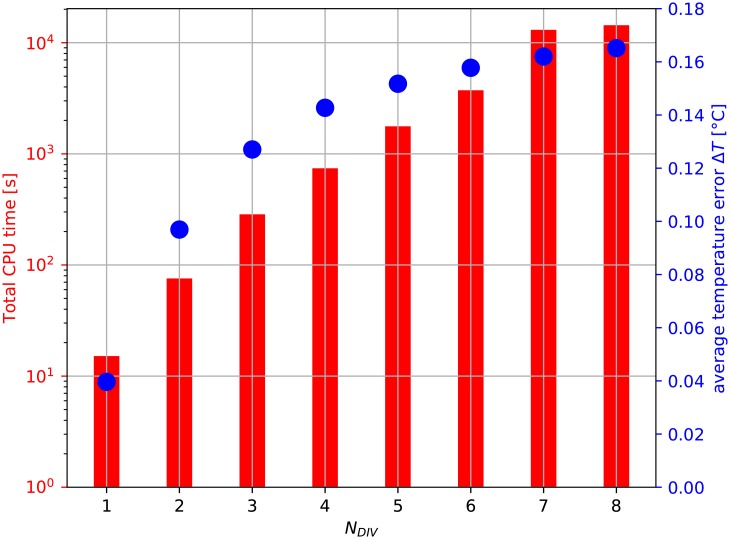
Influence of the finite element size (described by number of element divisions per line *N*_*DIV*_) on average temperature error Δ*T* and computation time.

To analyse the influence of the element size on IHTP accuracy, calculated heat flux was used to solve direct heat transfer problem for FEM models of various element sizes (*N*_*DIV*_ between 1 and 8). Results presented in Table 5 and Fig 9 show that for *N*_*DIV*_ > 1 require substantially more computation time. However, heat flux calculated for *N*_*DIV*_ = 1 provides good correspondence between calculated **T** and measured temperature **Y**.

**Table 5:** Comparison of calculations for various finite element sizes.

*N*_*DIV*_	total computation time [s]	memory used [MB]	nodes	elements
1	15.136	14.0	667	102
2	75.569	53.0	4268	816
3	285.286	175.0	13251	2754
4	740.645	251.0	30064	6528
5	1768.496	531.0	57155	12750
6	3729.508	1006.0	96972	22032
7	13059.517	1788.0	151963	34986
8	14373.839	2869.0	224576	52224

Also, using the same simulation results, in location of smallest finite element, the stability of the numerical calculations was verified. The temperature distribution **T**_**smallest**_ at this point was presented in Fig 8. This curve shows no signs of instability, which validates the accuracy of future calculations, even though the Fourier number criterion was not satisfied.

**Fig 10:**
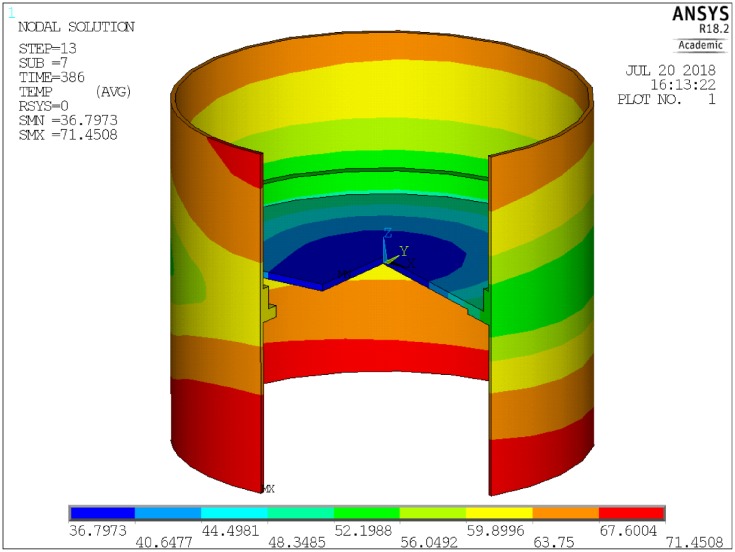
Temperature distribution.

**Fig 11:**
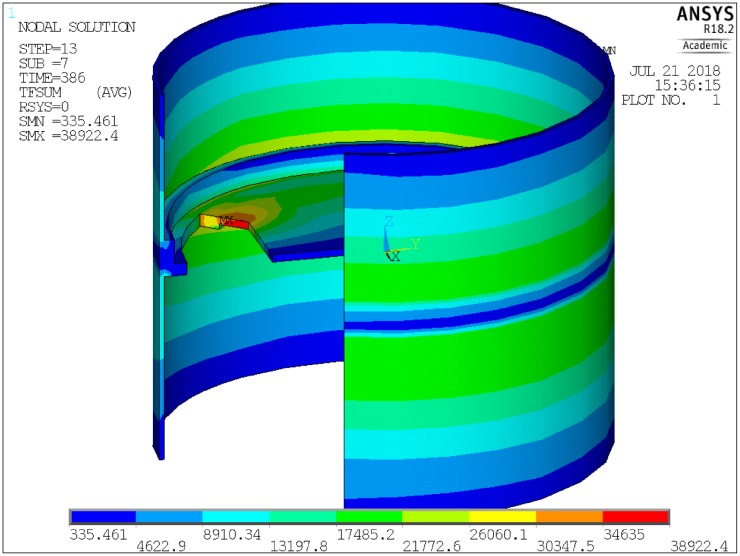
Heat flux distribution.

Similarly, heat flow through the smallest finite element **q**_**smallest**_ was presented in Fig 8. This curve is also smooth, further validating the approach. The distribution of temperature was presented in Fig 10, and the distribution of heat flux was presented in Fig 11.

**Fig 12:**
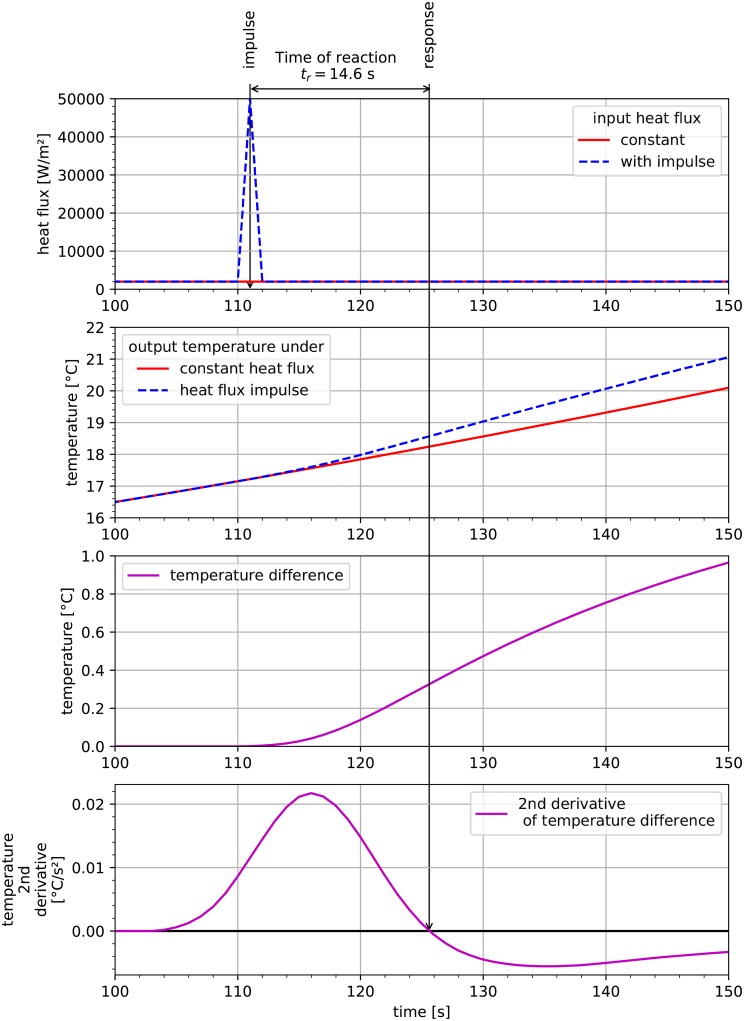
Determining the reaction time of the studied system.

Additionally, a test was performed to check the time of reaction to rapid change in the boundary conditions. After some time of stabilization, a sudden, short increase in heat flux was applied. For reference, a constant heat flux profile was generated (see Fig 12). The temperature outputs of the two cases was calculated and presented below. Furthermore, the temperature difference between these two time profiles was calculated to determine the time lag. A second derivative of the temperature difference was calculated. Its root was determined as the border moment between the progressive and degressive regime. This moment is the apogee of the impulse action.

A time lag of ≈ 14.6 s was observed. This can be interpreted as a minimal time difference to observe the results of heat flux change in the system.

### 2.3 IHTP calculations

#### 2.3.1 Goal function and decision variables in the optimization procedure

The calculations revealed spatial and temporal temperature distribution in the model. The values of temperature *T*_*j*_ measured at the point shown in Fig 1 were calculated so that they can be compared with measured values *Y*_*j*_. **T** and **Y** are both discretized in *N* samples with the corresponding vector of timestamps **t**.

The temporal profile of heat flux density **q** was modified so that the resulting calculated temporal profile **T** approached the measured values **Y**. Accuracy was measured using formula 1 as a sum of the differences in each second and is used as a goal function in the optimization procedure. Average temperature error was calculated using modified formula 1 as:

$$\begin{array}{c} \Delta T=\frac{\sum_{j=1}^{N}\left|Y_{j}-T_{j}\right|}{N} \end{array}$$ (7)

The goal function, thus defined is subject to minimization in the course of the optimization procedure. This is achieved by successive corrections of decision variable vector **q**, which contains discretized values of heat flux in *n* time spans **s**, so that the heat flux value *q*_*i*_ is applied throughout time span *s*_*i*_. Since optimization of *N* variables (as *N* is the number of measurement samples **Y**) would result in an unreasonably lengthy computation, *n* ≪ *N* was chosen (choice of *n* is described in subsection 2.3.3). During FEM transient heat flow analysis, its continuity was recreated via linear interpolation between time steps across the time span performed by ANSYS.

#### 2.3.2 Moving window optimization

Moving window optimization is a novel optimization approach for finding the temporal profile of inputs given the temporal profile of the output. Authors Quental et al. [29] applied it for calculating inverse dynamics for biomechanics of motion. In this work, we propose to apply this technique to solve the inverse heat transfer problem (IHTP).

**Fig 13:**
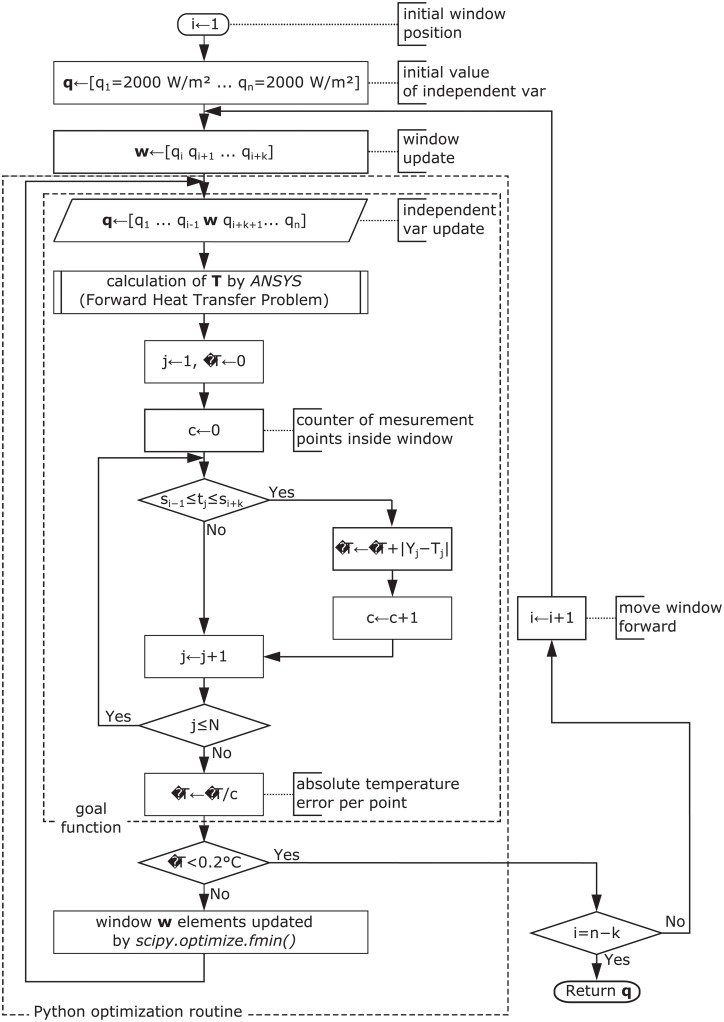
Moving window optimization flowchart.

**Fig 14:**
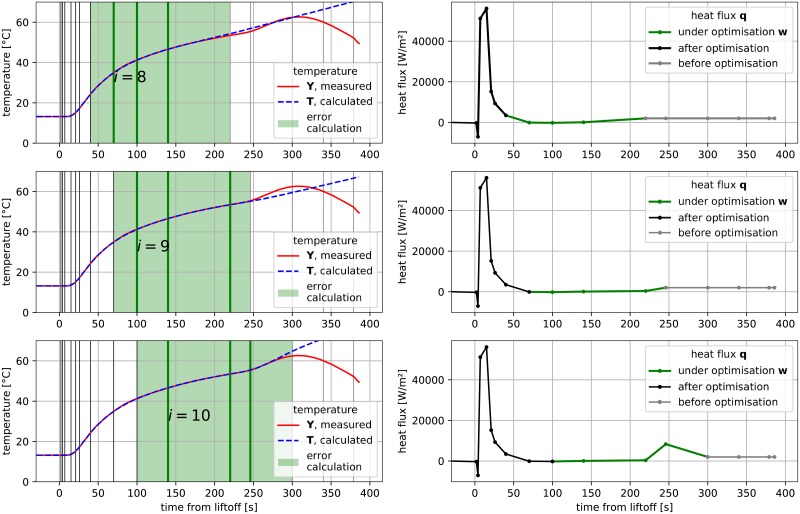
**Moving window optimization Left: Visualization of three consecutive positions** (*i* = 8—**upper**, *i* = 9—**middle**, *i* = 10—**bottom**) **of moving window for** *n* = 16, *k* = 3. **Right (corresponding to left): linear interpolation of heat flux q values is visible. Points indicate time spans s**.

The main idea of the algorithm is to use a temporal moving window of *k* < *n* time spans and performing optimization only on a limited vector **w** ⊂ **q** for time spans between *s*_*i*_ and *s*_*i*+*k*−1_ rather than the whole domain of heat flux values **q** and time spans **s**. Additionally, the goal function is evaluated only on a limited number of samples that have a time stamp *t*_*j*_ that satisfies criterion *s*_*i*−1_ ≤ *t*_*j*_ ≤ *s*_*i*+*k*_. This allows only temperature values influenced by heat flux vector **w** to be taken into consideration, while simultaneously ignoring temperature values that were significantly earlier or later. In the process of optimization, these time spans can be treated as *n* dimensions in multidimensional minimization. When this process finishes, the first dimension is removed and the next dimension is added at the end of the window, so that eventually the window processes the whole domain (i.e. all dimensions). The flowchart of the moving window optimization process is presented in Fig 13. Note that the stop criterion (Δ*T* ≤ 0.2) is extended in implementation to avoid situations where minor changes of **w** insignificantly influence goal function value (change in any element of **w** between iterations ≤ 20 *W*/*m*^2^). A visualization of three consecutive positions of the moving window is presented in Fig 14.

#### 2.3.3 Investigation of algorithm parameters

This algorithm is only an improvement to a traditional optimization procedure. As such, it requires a different optimization procedure. In the case of IHTP, minimization of Eq (1) was performed using Nelder-Mead simplex algorithm [30] implemented using Python in the SciPy library [31].

The advantage of the moving window algorithm is that it transforms *n*-dimensional optimization into a series of *k*-dimensional optimizations. This allows the number of goal function evaluations to be significantly decreased, and therefore the computing time (see Table 4). Another advantage is that shorter time segments are optimized in order to match cause (heat flux) with effect (temperature change) more precisely.

**Fig 15:**
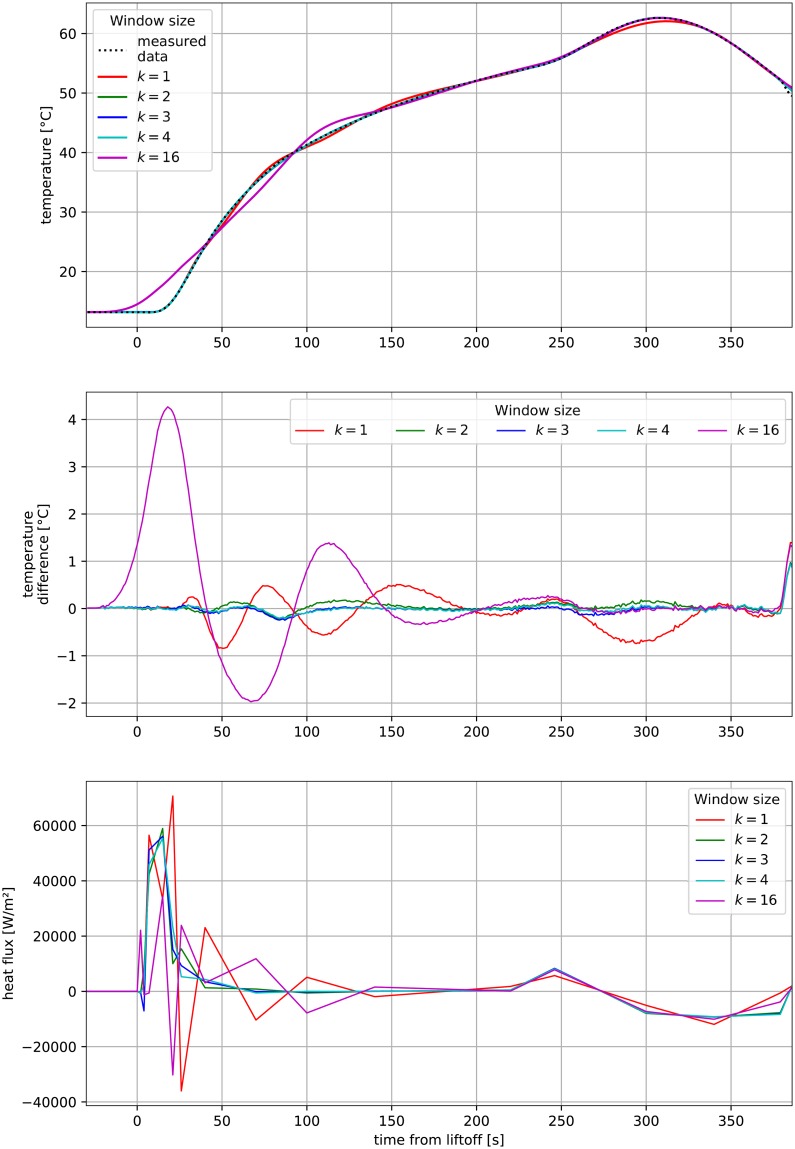
Comparison of test optimization results for with various moving window lengths *k*.

A common problem in multidimensional optimization is that some dimensions of input (e.g. heat flux at the end of the flight) may have little to no effect on some parts of the output (temperature change at the beginning of the flight). Also, typically a great number of decision variables poses a problem for optimization in that multiple inaccurate solutions arise at the early stages of resolving [32]. The next stage of refining of the solution leads to one of many local minima, which, for decision variables that can be interpreted as consecutive points in a time series, eventually leads to oscillatory behavior. This oscillation feature of heat flux can almost perfectly recreate the measured temperature profile, although it has no physical interpretation. This is clearly visible when the window was designed to cover all *n* time spans (see Fig 15, for *k* = 16). In this case, the proposed algorithm becomes equivalent to typical optimization, without a moving window (*k* = *n*). A comparison of the number of total goal function iterations and errors per point Δ*T* for various window lengths *k* was presented in Table 4, with *k* = 4 chosen as the best and used in subsequent sections.

**Fig 16:**
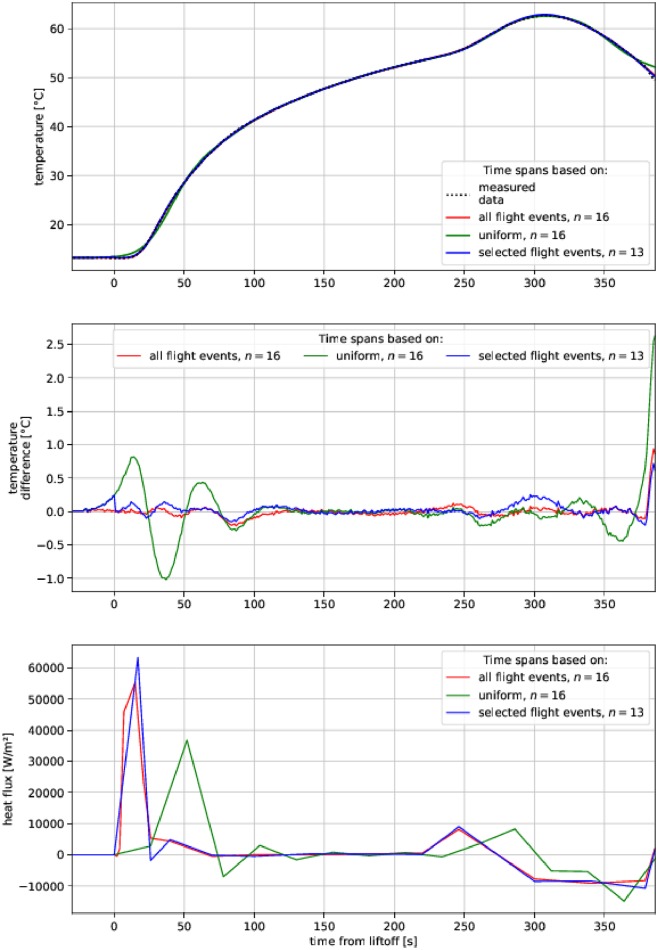
Comparison of test optimization results with various division of heat flux q into time spans s. **All calculations were performed with window length** *k* = 4.

Further studies of the method showed that temporal span distribution is crucial for the application of this method in IHTP (see Fig 16). Correlating interpolation points (i.e. time spans **s**) of heat flux **q** with important events during rocket flights (see Table 1) provided substantial improvement to the results. This proved to be much better than uniform timespan distribution, which resulted in heat flux **q** oscillations. The proper distribution of time spans **s** affects solution accuracy more than the number of time spans *n*: the selected 13 spans provided better results than a uniform 16. Decreasing the number of points was achieved by removing three quick events at the beginning of the flight. This, however, resulted in a noticeable oscillation of heat flux **q** in the temporal vicinity of the removed events.

The optimization window **w** cannot be shorter than the reaction time *t*_*r*_ (see Fig 12). This is clearly visible in Fig 15 showing the results of optimization for *k* = 1. In this case, the time difference between the goal function Δ*T* calculation span and currently optimized heat flux value *q*_*i*_ is significantly less than *t*_*r*_ = 14.6 s (specifically: 4 s, 5 s, 11 s). As a result, changes to the window **w** do not substantially affect calculated temperature *T*. The optimization process is then inaccurate and the final calculated temperature *T* exhibits unjustified oscillatory behavior (see Fig 15 for *k* = 1). With such a short window **w**, the optimization procedure was not able to reach the required criterion Δ ≤ 0.2°*C* (see Table 4). This was only possible for a longer window, e.g. *k* = 3 or *k* = 4, for which the shortest sum of spans **s** within window **w** were: 15 s and 19 s, respectively.

A conclusion can be drawn that in order to achieve precise interpolation of heat flux **q**, corresponding time spans **s** must be short (with dynamic systems, significantly smaller than time of reaction *t*_*r*_). Simultaneously, the optimization procedure should observe the system reaction (via calculating goal function) in steps longer than *t*_*r*_. A trivial solution would be calculating goal function for the whole process time (*t*_*flight*_), however this leads to inaccurate results and requires significant computational powers. **A method for reconciling these conflicting requirements for the time step is the *moving window* optimization**.

## 3 Results & discussion

**Fig 17:**
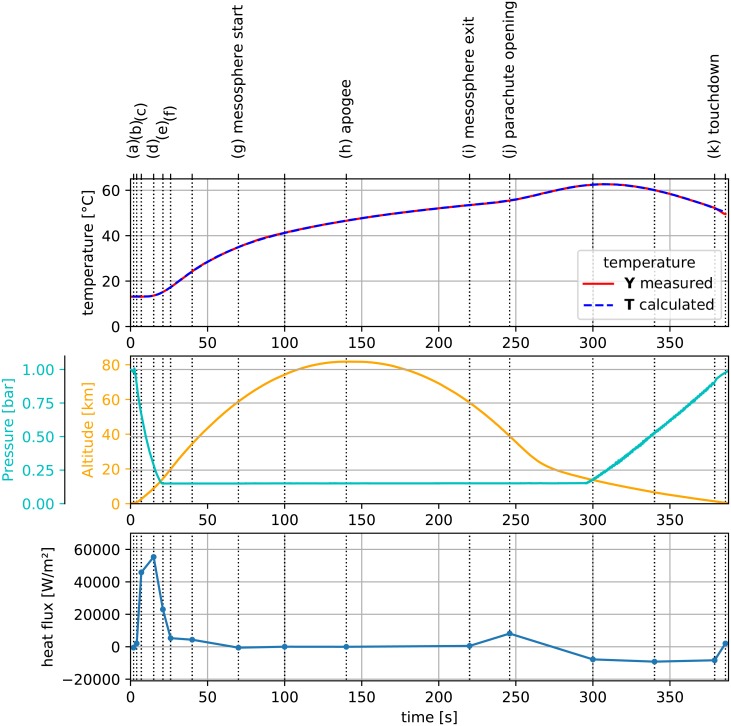
Calculated heat flux q and temperature T against variables measured during flight: Temperature Y (used for IHTP), pressure, altitude. Vertical lines indicate characteristic phases of flight, see Table 1 for details.

Fig 17 shows calculated heat flux **q** against variables measured during flight. Time spans **s** distribution was based on characteristic phases of flight, listed in Table 1 (*n* = 16). Moving window size was chosen as *k* = 4. A rapid peak in heat flux **q** is visible during motor burn up to a value of 60000 *W*/*m*^2^. As the rocket gains altitude, the density of the air it traverses decreases. This is visible in the first three segments of the heat flux **q** profile. First stage describes the phase of flight during which the rocket has not reached high velocity yet, thus resulting in a small heat flux. During the second stage, the rocket flies through thick layers of atmosphere with substantial velocity, thus generating the greatest value of heat flux. In the stage, shortly before and after motor burnout (see Fig 2), a small heat flux is still present, presumably due to rapid ascent through thick layers of air of decreasing density. As the rocket reaches the mesosphere, heat transfer is significantly reduced due to a lack of exchange medium (very low pressure). This would be visible in pressure data; however the pressure sensor range preculded the measurement of low pressure in the mesosphere. The heat flow only increases as the rocket egresses the mesosphere and enters the stratosphere. The rapid descent is then decelerated by opening parachutes. As the payload traverses the stratosphere at a steady pace, presumably the forced convection to colder air reverses the flow of heat. This is visible in Fig 17 as a negative value of heat flux.

Calculated temporal profile of heat flux **q**, using the experiment measurements, correlates well with the characteristic phases of flight. However, the analysis in the the method showed great sensitivity to parameter values (different number *n* of time spans **s**, their distribution unrelated to flight events, length *k* of moving window **w**). Small changes in the above-mentioned parameters affect the calculated heat flux **q** vastly, regardless of the correspondence between calculated temperature profile **T** with measurement values **Y**. An Additional source of uncertainty is the assumed value of heat transfer coefficient *h* of convection at dropping pressure (see Section 2.2.2).

## 4 Conclusions

The heat inverse problem solution presented in this paper resulted in estimation of heat flux at the surface of a sounding rocket. Results from actual sounding rocket flight are used. Classic IHTP methods modified slightly using modern optimization and FEM approaches are applied. Measurements from an actual rocket flights are used. Methods presented here can be used in future engineering calculations.

**Fig 18:**
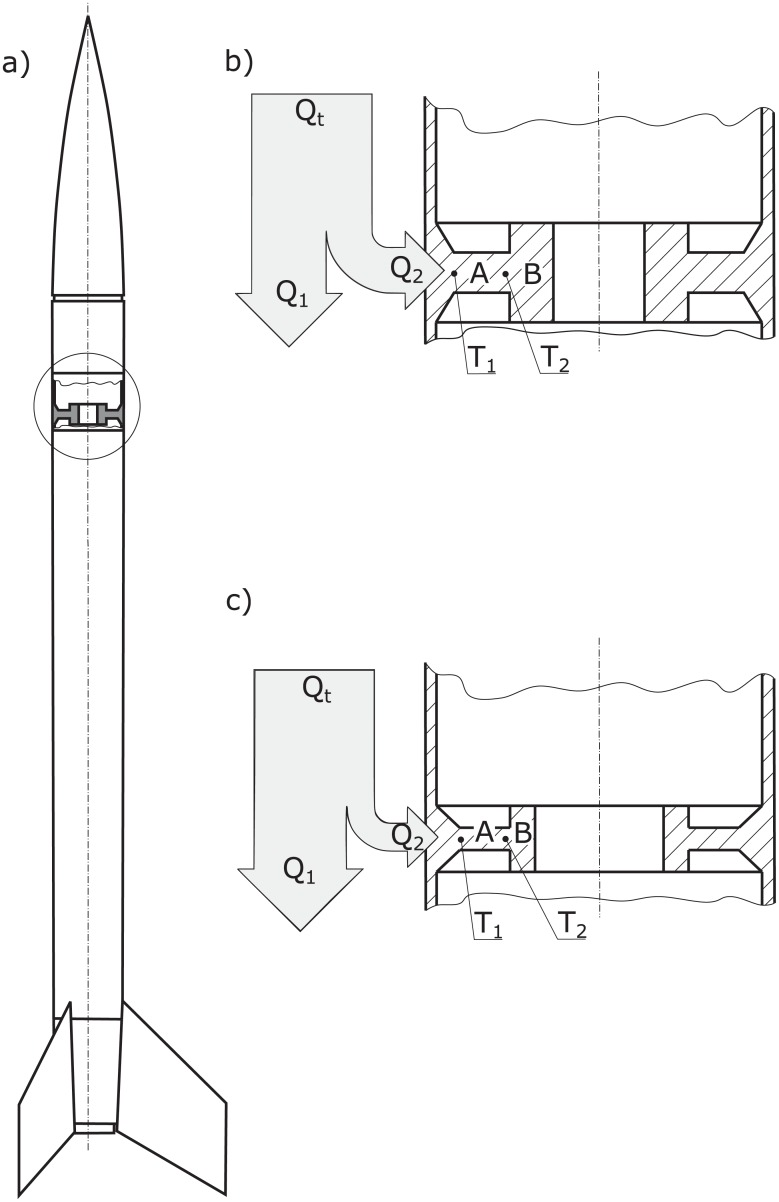
Schematic view of the planned experiment to measure heat flux on sounding rocket skin. Note that insulation was not shown here.

A new sounding rocket experiment has been designed to resolve these problems and further investigate the topic of sounding rocket heat transfer. The idea of the experiment is presented in Fig 18. Its aim is to measure the heat flow through a resistive element (*A*) to the heat container (*B*). during the course of the entire flight. The heat capacity of container B was designed to be enough to ensure measurable temperature gradient between points *T*_1_ and *T*_2_, providing accurate and immediate information on heat flow *Q*_2_. This avoids speculative IHTP and heat flow is enforced by known constant heat capacity of container B rather than unknown convection in the environment of rapidly changing air pressure as the rocket reaches high altitudes. The measurement system will be matched with a CFD model of the rocket. This is necessary as the heat *Q*_*t*_ generated on rocket skin splits into heat flowing to the inside of the rocket *Q*_2_ and heat transported with air flow around the vehicle *Q*_1_. The fraction $\frac{Q_{2}}{Q_{1}}$ highly depends on heat resistance of element (*A*) and heat capacity of container (*B*)—compare Fig 18b) and 18c).

The experiment is scheduled for flight on REXUS25 rocket in March 2019 from Esrange Space Center in Kiruna, Sweden within ESA REXUS/BEXUS program [16]. The device is patent pending in Poland Patent Office, application number *P.423198*.

All data underlying the findings in this paper are available in repository [33].

## Abbreviations

BEXUS: Balloon EXperiment for University Students programme
CFD: Computational Fluid Dynamics
ESA: European Space Agency
FEM: Finite Element Method
IHTP: Inverse Heat Transfer Problem
NTC: Negative Temperature Coefficient thermistor
REXUS: Rocket EXperiment for University Students programme
SMARD: Shape Memory Alloy Reusable Deployment Mechanism experiment
TU: Technical University
